# Supplementary material for: Retinoic Acid Fluctuation Activates an Uneven, Direction-Dependent Network-Wide Robustness Response in Early Embryogenesis
Source: Front Cell Dev Biol. 2021 Oct 20;9:747969. doi: 10.3389/fcell.2021.747969 (PMC8564372; doi:10.3389/fcell.2021.747969)
Supplement: Supplementary file 1 [file Data_Sheet_1.pdf]

Parihar et al.
Supplemental Figure S1

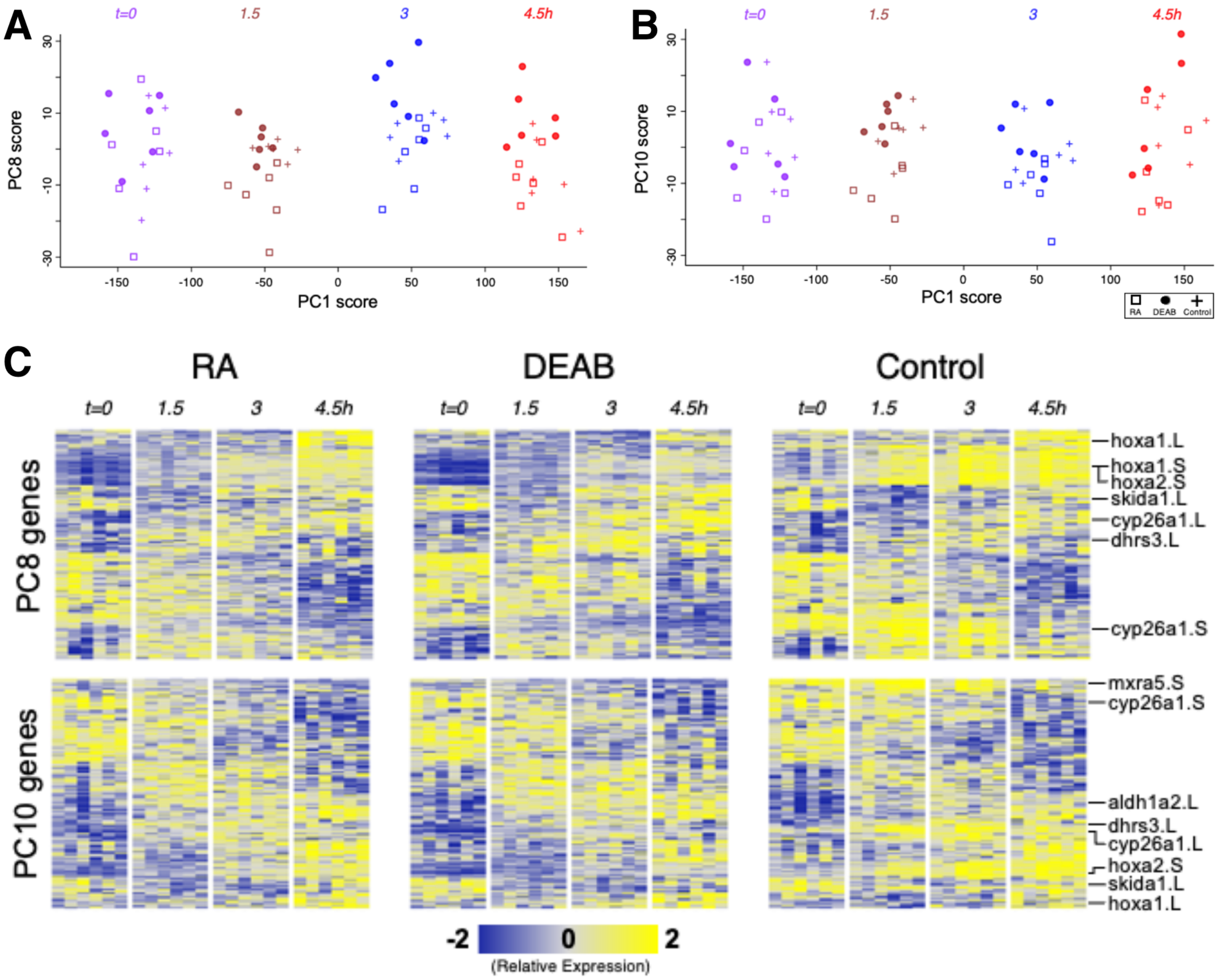

Supplemental Figure S1. Efficient RA signaling robustness at the transcriptomic level. (A,B) Principal Component Analysis of six clutches over time for **(A)** PC1/PC8, and **(B)** PC1/PC10. **(C)** Heatmap of gene expression of the top-100 positive and top-100 negative loadings corresponding to PC8 and PC10. A subset of the genes is highlighted based on the relevance to early RA metabolism.

Parihar et al.

Supplemental Figure S2

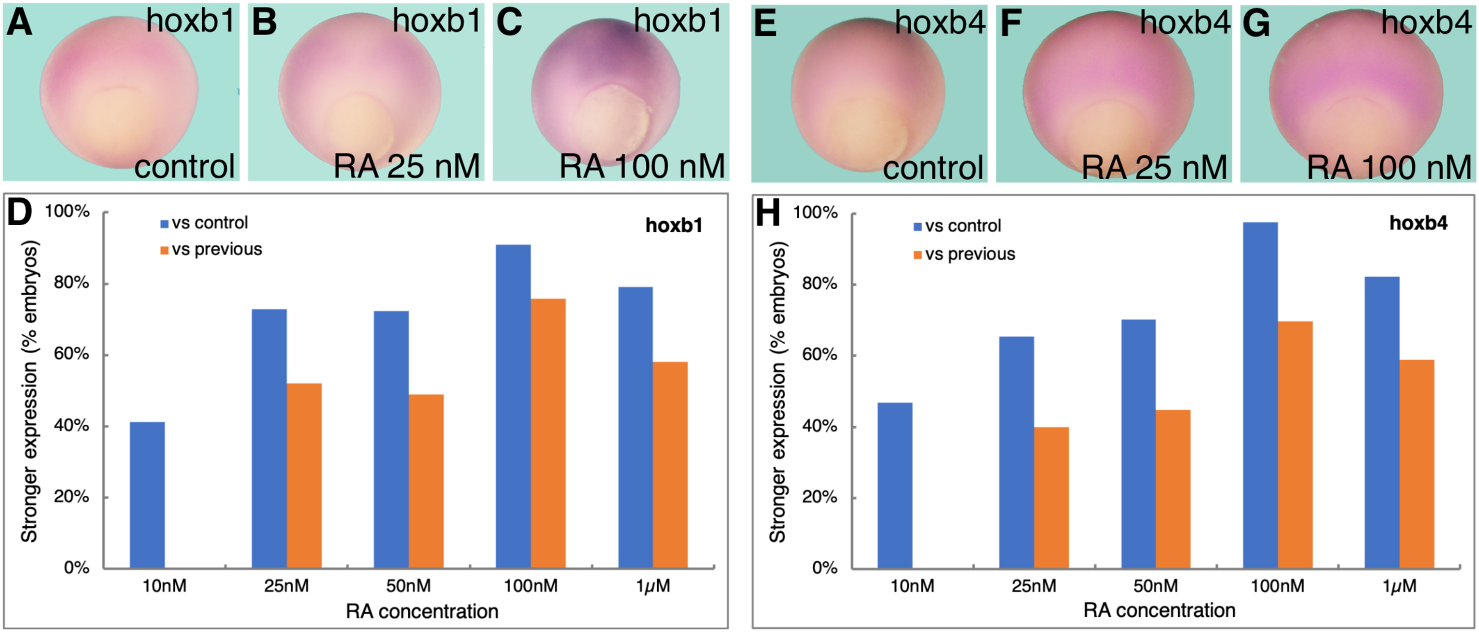

Supplemental Figure S2. Dose response of *hoxb1* and *hoxb4* to increased retinoic acid levels. Embryos were treated with increased retinoic acid concentrations (10 nM - 1 μM) until mid gastrula (st. 11) and then processed for *in situ* hybridization with probes specific for *hoxb1* **(A-C)** or *hoxb4* **(E-G)**. **(D,H)** The changes in the expression pattern for both genes was scored as an increase in signal intensity from the control group (vs. control) or the previous lower concentration (vs. previous).

Parihar et al.
Supplemental Figure S3

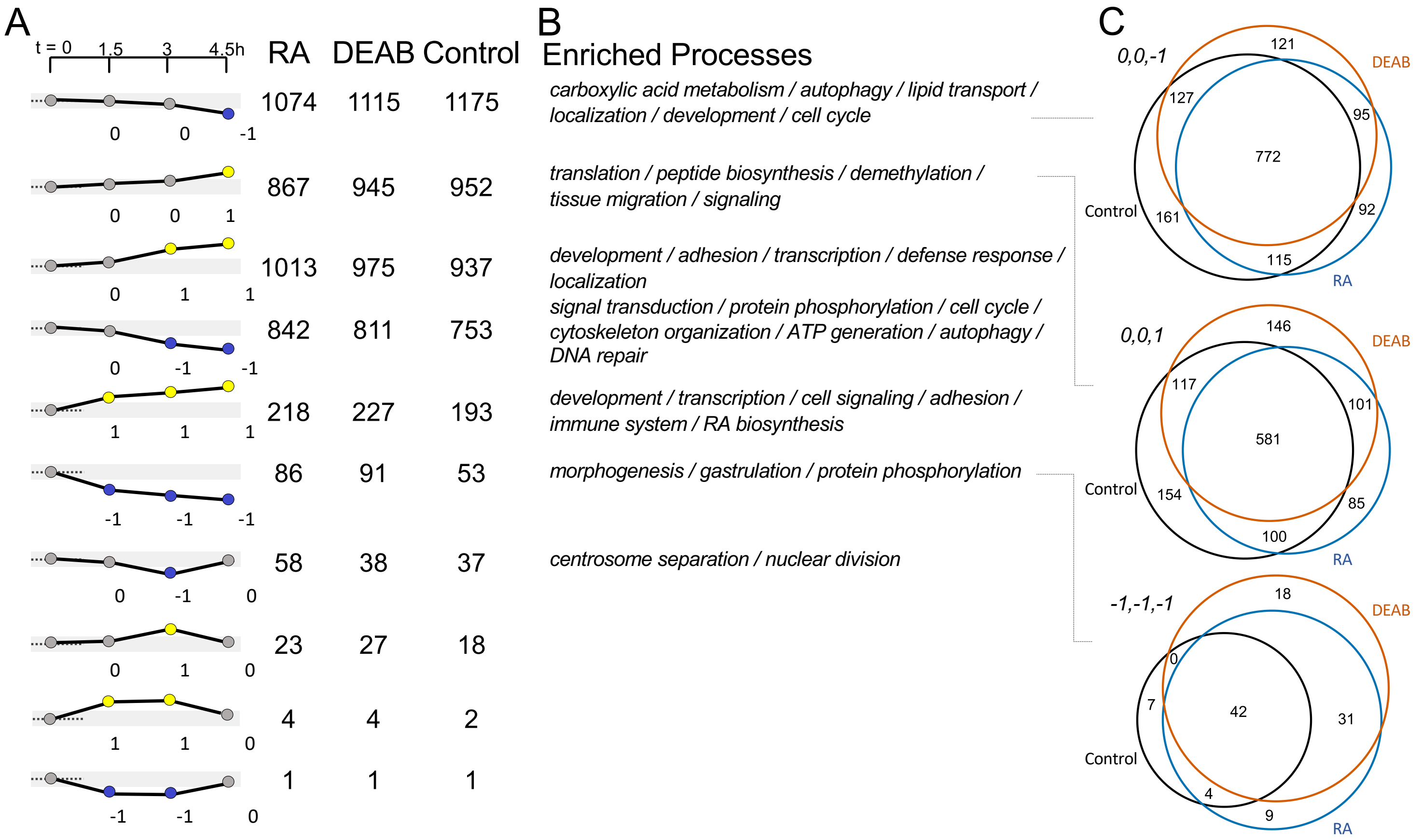

Supplemental Figure S3 - Transcriptome overlap between RA manipulation and controls. (A) Genes were grouped into the 27 possible discretized expression patterns based on up-regulation (yellow), down-regulation (blue), and no change (grey) above the 2-fold change threshold at each of the three recovery time points compared to the t=0 sample. The ten dynamic patterns that contained at least one gene are shown. The numbers below each graph exemplify the discretized pattern as a numeric vector (+1, 0, -1). The counts next to the patterns indicate the number of genes that show the corresponding expression pattern in each of the two treatment groups and the controls. **(B)** The Gene Ontology biological processes statistically enriched in the control group are indicated alongside the pattern counts. Details of statistical analysis results are available in Supplemental Table S1. **(C)** Venn diagrams to compare the overlap between the two treatments and control, illustrated for three differential gene expression patterns. The number of genes that showed similar differential expression patterns in one or more experimental groups are indicated in the corresponding overlapping regions. black - control; orange - DEAB; blue - RA.

Parihar et al.
Supplemental Figure S4

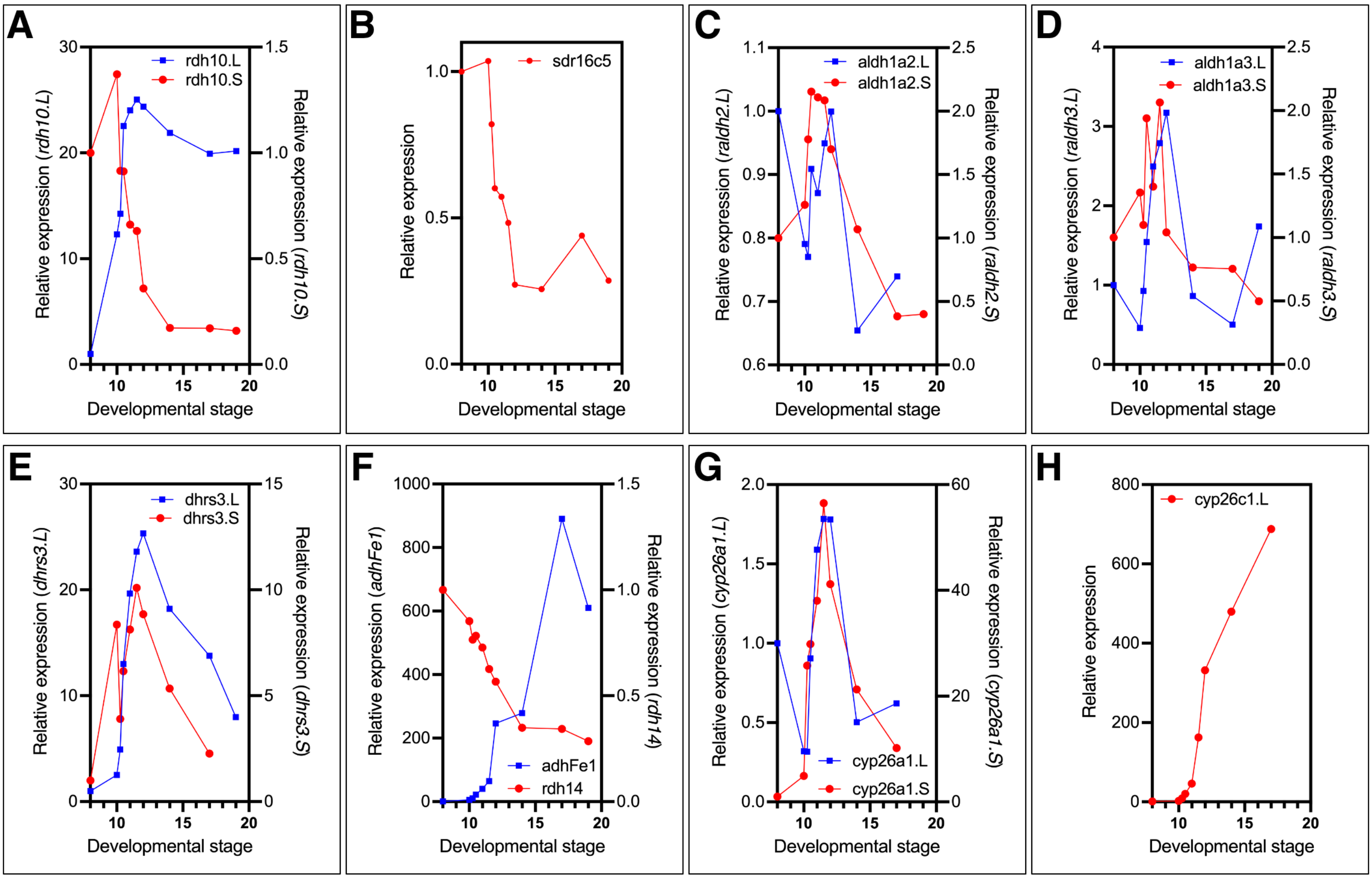

Supplemental Figure S4. Temporal expression pattern of the main RA network components studied. To determine the temporal pattern of expression of the RA network components, RNA samples from embryos from midblastula (st. 8) to advanced neurula stages (st. 19) were collected. Relative expression was determined by qPCR for: **(A)** *rdh10.L, rdh10.S*; **(B)** *sdr16c5*; **(C)** *aldh1a2.L, aldh1a2.S*; **(D)** *aldh1a3.L, aldh1a3.S*; **(E)** *dhrs3.L, dhrs3.S*; **(F)** *adhFe1, rdh14*; **(G)** *cyp26a1.L, cyp26a1.S*; **(H)** *cyp26c1.L.*

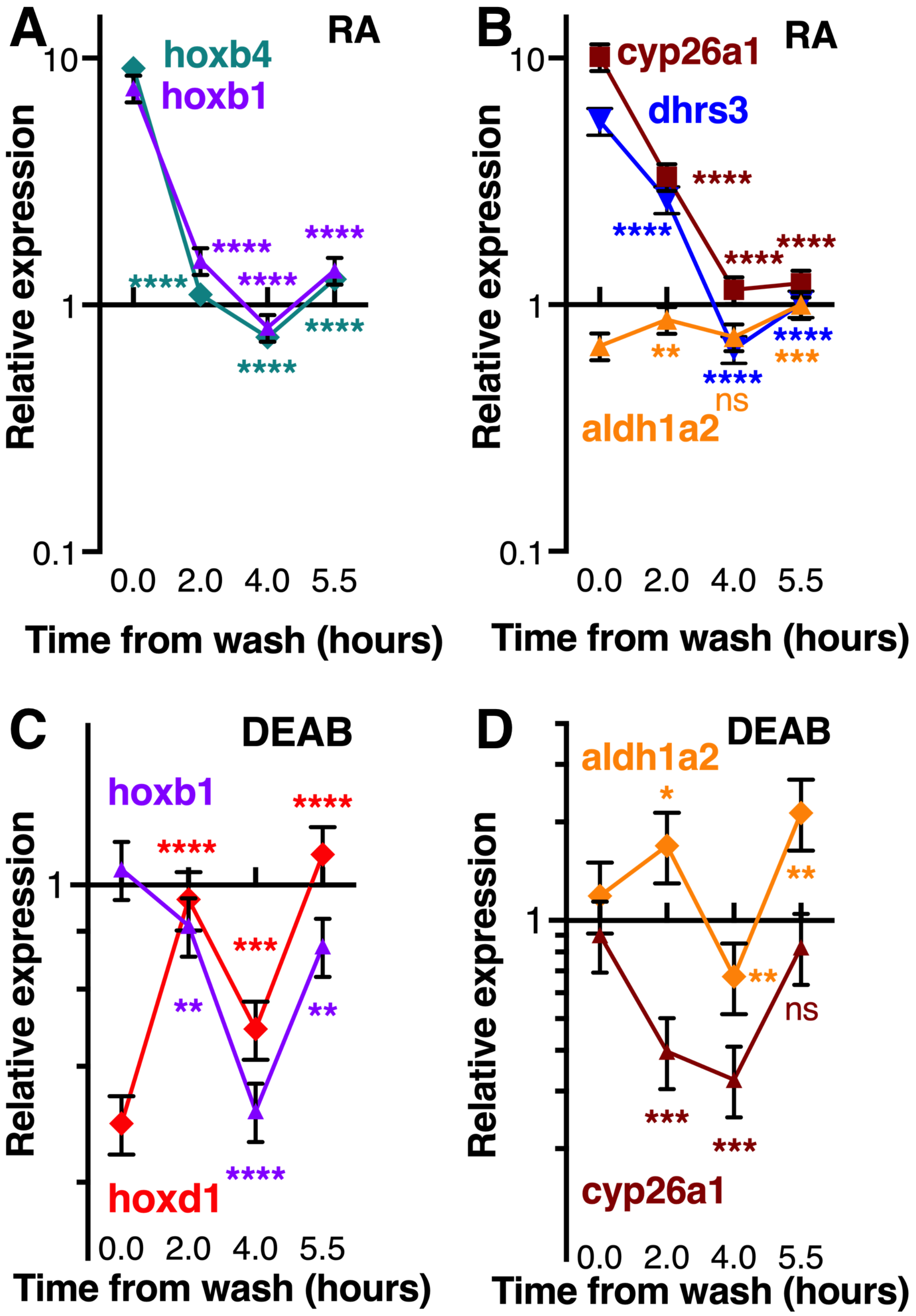

Supplemental Figure S5. Kinetics of the recovery from RA manipulation. Embryos were transiently treated with either 10 nM RA **(A,B)**, or 50 µM DEAB **(C,D)**. Treatments were initiated during late blastula (st. 9.5), and washed by early gastrula (st. 10.25). RNA samples were collected at different time points during the recovery period. The response of genes, RA target, and RA metabolic enzymes was studied by qPCR. Statistical significance (Student's t-test) was calculated compared to the expression at the end of the treatment (t0). *, p<0.05; **, p<0.01; ***, p<0.001; ****, p<0.0001; ns, not significant.

Parihar et al.
Supplemental Figure S6

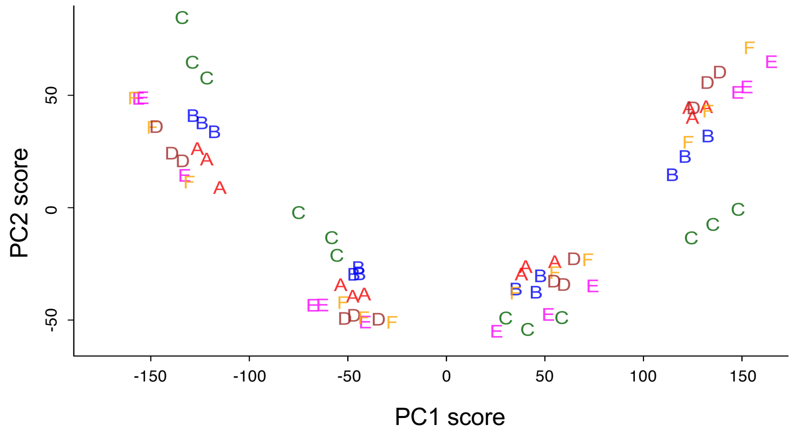

Supplemental Figure S6. Principal Component Analysis suggests clutch-to-clutch variation. Labeling of the different clutches reveals clustering of all samples of the same clutch at all time points analyzed.

Parihar et al.
Supplemental Figure S7

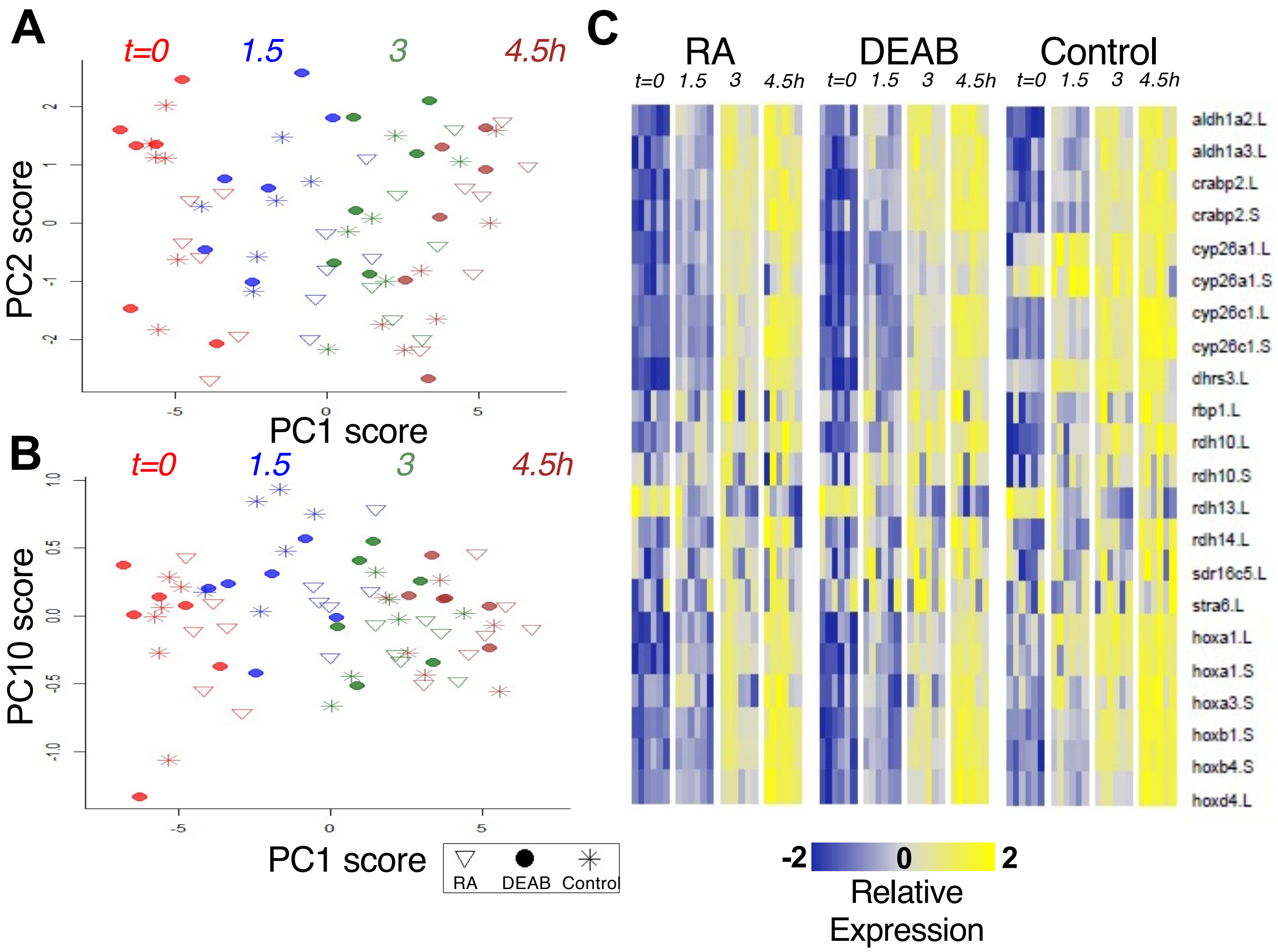

Supplemental Figure S7. HT-qPCR analysis of RA network components. Principal Component Analysis of the HT-qPCR data revealed heterogeneity across developmental stages and treatments. **(A)** Distribution of samples along PC1 and PC2 axes. **(B)** Distribution of samples along PC1 and PC10 axes. **(C)** Heatmap of time series differential expression of RA network genes.

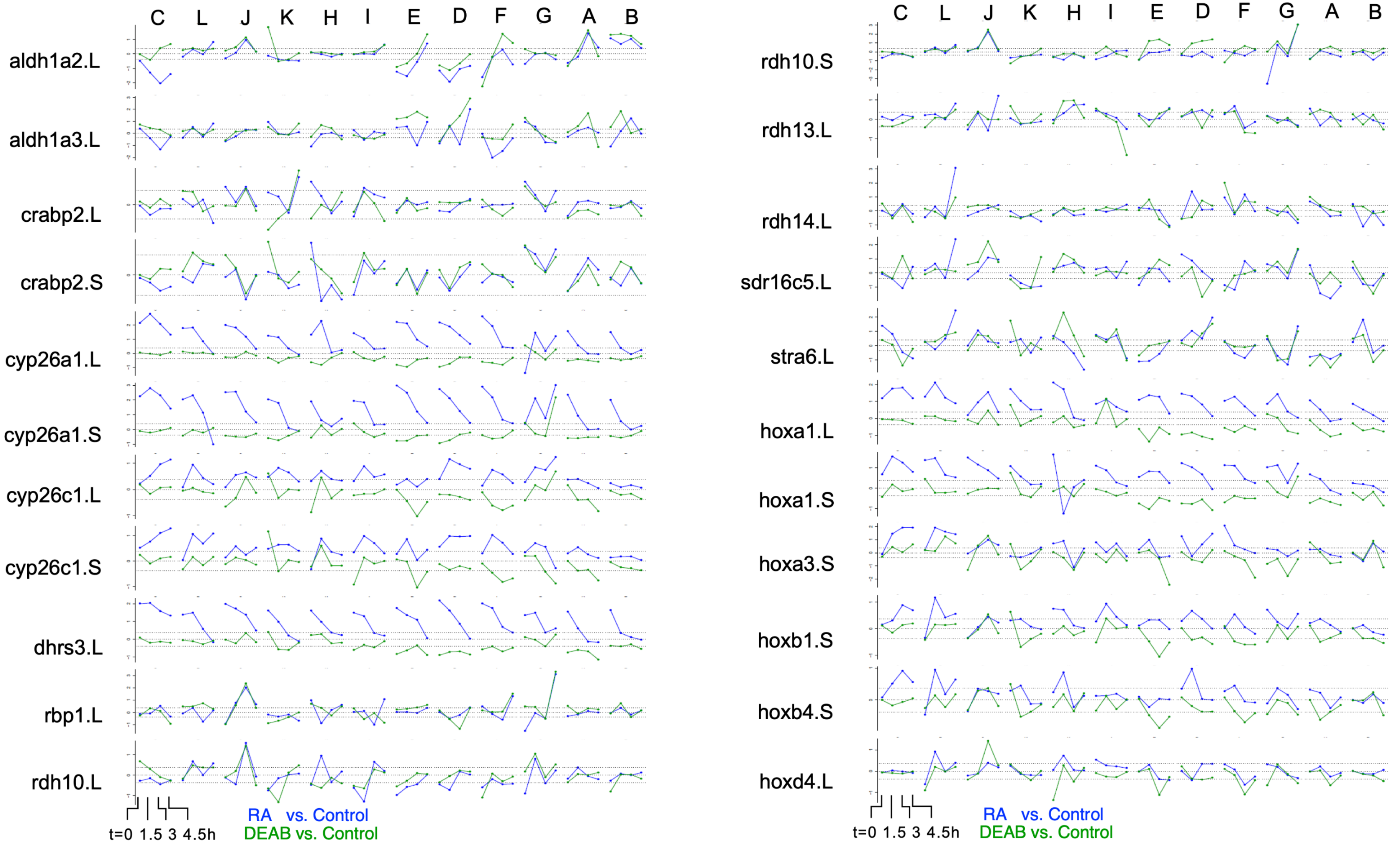

Supplemental Figure S8. Clutch-wise heterogeneity in the response of RA network genes following RA manipulation. Clutch-wise differential expression dynamics of RA network genes based on the RNAseq and HT-qPCR data. The clutches are ordered left to right according to the time taken for recovery of *hoxa1.L* expression.

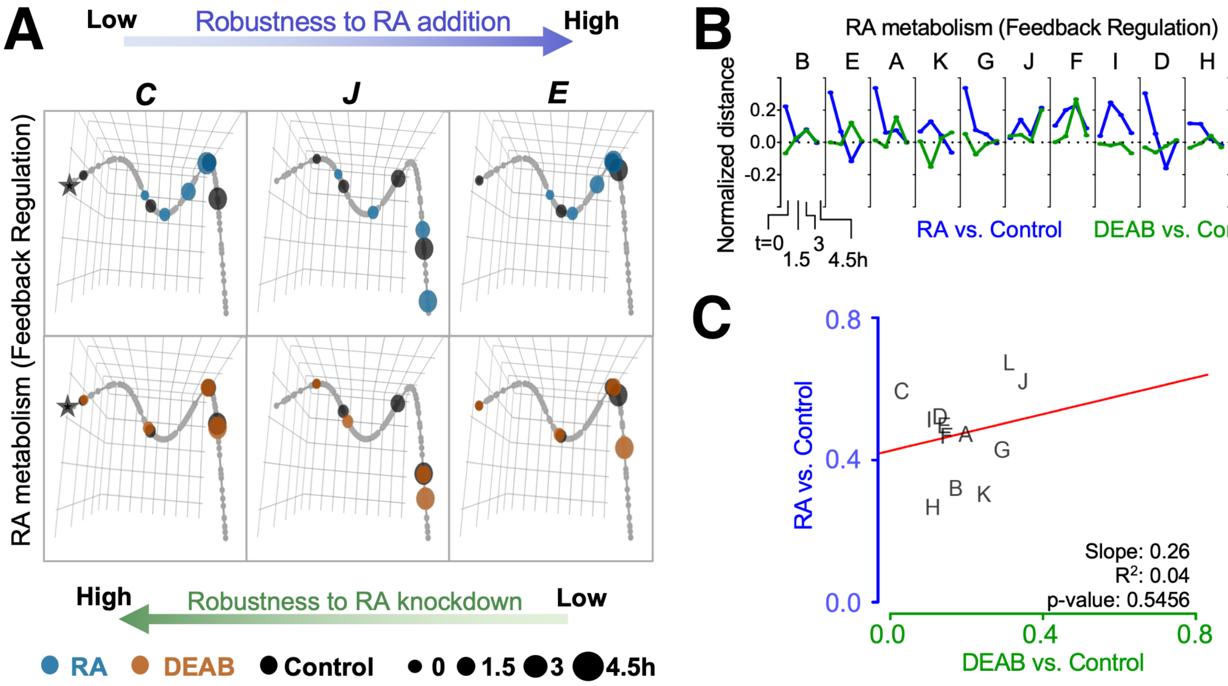

Supplemental Figure S9. Trajectory analysis to compare the extent of individual clutch robustness based on RA network component expression. (A) 3-dimensional principal curve for the RA network genes, showing projections of the sample points on the curve for **(A, top)** RA and **(A, bottom)** DEAB treatments. Principal curves for clutches C, J, and E are shown. The black star indicates the beginning of the curve for the distance measurement along the trajectory. Ranking of clutches is based on the net (absolute) normalized distance of treatment samples from the corresponding Control sample for each time point. **(B)** Normalized expression shift profile calculated from the principal curve as the arc distance between the treatment and the corresponding control. Clutches are rank-ordered from lowest to highest net expression shift for *hox* genes in the RA group. Clutches A-F data from RNA-seq, clutches G-L data from HT-qPCR. **(C)** Distribution of clutches based on the trajectory determined robustness to RA and DEAB treatments relative to each other based on the RA network genes. The letters indicate the distinct clutches.

Supplemental Table S1. List of Gene Ontology annotations with corresponding genes and statistical significance corresponding to Supplemental Figure S3.

Control Pattern	ID	Description	Total Genes	Pathway Genes	p value	adjusted q value
**0 0 0 -1**	GO:0046626	regulation of insulin receptor signaling pathway	426	5	2.90E-06	3.99E-03
	GO:0006914	autophagy	426	14	7.65E-05	1.50E-02
	GO:0006631	fatty acid metabolic process	426	11	1.45E-04	2.24E-02
	GO:0006096	glycolytic process	426	7	5.28E-04	3.80E-02
	GO:0008354	germ cell migration	426	3	7.55E-04	4.02E-02
	GO:0009799	specification of symmetry	426	5	1.04E-03	4.74E-02
	GO:0010876	lipid localization	426	13	1.07E-03	4.74E-02
**0 0 0 1**	GO:0006518	peptide metabolic process	302	44	5.82E-13	2.24E-10
	GO:0001708	cell fate specification	302	4	2.34E-03	2.11E-01
	GO:0060485	mesenchyme development	302	7	3.95E-03	2.68E-01
	GO:0090130	tissue migration	302	3	8.10E-03	4.42E-01
	GO:0010631	epithelial cell migration	302	2	8.83E-03	4.42E-01
**0 0 1 1**	GO:0048513	animal organ development	320	35	2.34E-08	1.95E-05
	GO:0007155	cell adhesion	320	26	3.21E-07	7.75E-05
	GO:0030334	regulation of cell migration	320	5	6.53E-03	7.35E-02
	GO:1905209	positive regulation of cardiocyte differentiation	320	2	9.88E-03	8.84E-02
**0 0 -1 -1**	GO:0007020	microtubule nucleation	250	3	3.01E-03	4.81E-01
	GO:0009967	positive regulation of signal transduction	250	9	5.54E-03	4.81E-01
	GO:0098927	vesicle-mediated transport between endosomal compartments	250	2	6.11E-03	4.81E-01
	GO:0010256	endomembrane system organization	250	7	6.13E-03	4.81E-01
**0 1 1 1**	GO:0048513	animal organ development	83	15	5.49E-07	1.25E-04
	GO:0048732	gland development	83	5	1.42E-06	1.25E-04
	GO:0010557	positive regulation of macromolecule biosynthetic process	83	11	3.29E-06	1.97E-04
	GO:0031328	positive regulation of cellular biosynthetic process	83	11	4.39E-06	1.97E-04
	GO:0016055	Wnt signaling pathway	83	8	2.71E-04	4.57E-03
	GO:0044344	cellular response to fibroblast growth factor stimulus	83	4	4.94E-04	6.21E-03
	GO:0010817	regulation of hormone levels	83	4	1.92E-03	1.84E-02
	GO:0042572	retinol metabolic process	83	2	2.43E-03	2.28E-02
**0 -1 -1 -1**	GO:0008285	negative regulation of cell population proliferation	15	3	7.57E-05	6.61E-03
	GO:0007369	gastrulation	15	2	5.21E-03	9.93E-02
**0 0 -1 0**	GO:0007100	mitotic centrosome separation	14	2	1.87E-05	2.16E-03
	GO:0140014	mitotic nuclear division	14	2	5.71E-03	8.00E-02
	GO:0051128	regulation of cellular component organization	14	3	8.47E-03	8.00E-02
	GO:0031338	regulation of vesicle fusion	14	1	8.51E-03	8.00E-02
	GO:0032418	lysosome localization	14	1	9.92E-03	8.00E-02
**0 0 1 0**	GO:0045663	positive regulation of myoblast differentiation	4	1	2.84E-03	1.71E-02
	GO:0045445	myoblast differentiation	4	1	3.25E-03	1.71E-02

Supplemental Table S2. Weighted Gene Correlation Network Analysis (WGCNA)

Gene ID	Gene Names	Module Numbers	Module Colors	RA-CTRL pattern	DEAB-CTRL pattern
Xelaev18001038	LOC108703560	5	green	1_1_1_1	0_0_-1_-1
Xelaev18002824	sema3f.L	5	green	0_0_1_1	0_0_0_-1
Xelaev18006802	nfib.L	5	green	0_0_1_1	0_0_0_-1
Xelaev18007035	cmtm5.L	5	green	1_1_1_0	0_0_-1_-1
Xelaev18010181	tdgf1.2.S	5	green	0_1_1_0	0_0_0_-1
Xelaev18012533	LOC108708243	5	green	0_0_0_1	0_0_0_-1
Xelaev18013332	prph.L	5	green	1_1_1_1	0_0_0_-1
Xelaev18014991	hnf1b.S	5	green	0_1_1_0	0_0_-1_-1
Xelaev18015206	LOC108709115	5	green	0_0_1_0	0_0_0_-1
Xelaev18024402	pax6.S	5	green	0_0_1_0	0_0_0_-1
Xelaev18024482	LOC108715248	5	green	0_1_1_0	0_0_0_-1
Xelaev18025805	abhd14a.S	5	green	0_1_0_0	0_0_-1_0
Xelaev18030578	LOC108718689	5	green	0_1_1_0	0_0_-1_-1
Xelaev18030982	hoxa1.L	5	green	1_1_1_0	-1_-1_-1_-1
Xelaev18031638	gcnt2.L	5	green	0_0_0_1	0_0_-1_0
Xelaev18033067	hoxa1.S	5	green	1_1_1_0	-1_-1_-1_-1
Xelaev18033068	hoxa2.S	5	green	1_1_0_0	0_-1_-1_-1
Xelaev18033069	hoxa3.S	5	green	1_0_0_0	0_0_0_-1
Xelaev18033072	hoxa5.S	5	green	0_0_1_0	0_0_0_-1
Xelaev18034595	cyp26c1.L	5	green	0_1_1_1	0_-1_-1_-1
Xelaev18035730	dhrs3.L	5	green	1_1_1_0	-1_0_0_-1
Xelaev18036884	cyp26a1.S	5	green	1_1_1_0	-1_-1_0_0
Xelaev18036885	cyp26c1.S	5	green	1_1_1_1	0_0_-1_-1
Xelaev18037155	neurog3.S	5	green	0_1_0_0	0_0_0_-1
Xelaev18037556	LOC108697667	5	green	1_1_1_0	0_-1_0_0
Xelaev18038437		5	green	0_0_1_0	0_0_0_-1
Xelaev18039368_meis3.L	meis3.L	5	green	0_1_0_0	-1_-1_0_0
Xelaev18041724	LOC108699981	5	green	0_1_1_0	0_0_-1_-1
Xelaev18044027	LOC108701808	5	green	0_1_1_0	0_-1_0_0
Xelaev18044028		5	green	1_1_1_0	0_0_0_-1
Xelaev18044734	hoxd1.L	5	green	0_1_0_0	-1_0_0_0
Xelaev18045501	LOC100489456.L	5	green	0_0_1_0	0_0_-1_-1
Xelaev18045983	hoxb1.S	5	green	0_1_0_0	0_-1_-1_-1
Xelaev18047280	gbx2.1.L	5	green	0_1_1_1	0_-1_-1_-1

Supplemental Table S3. RA network component polymorphisms between *Xenopus* strains[1]

Gene name	*Gene ID*	*aa position*	*aa1*	*aa2*
aldh1a2	18020673m	240	P	S
cyp26c1	18034595m	388	F	I
rbp1	18027898m	26	T	I
rbp1	18027898m	149	E	K
rbp1	18027898m	27	H	R
rdh10	18033799m	133	H	R
rdh13	18026677m	14	C	F
sdr16c5	18032037m	35	A	T
stra6	18018036m	610	S	N

[1]based on (Savova et al., 2017)
